# Loss of In Vivo Replication Fitness of HIV-1 Variants Resistant to the Tat Inhibitor, dCA

**DOI:** 10.3390/v15040950

**Published:** 2023-04-12

**Authors:** Lijun Ling, Ana R. Leda, Nurjahan Begum, Rae Ann Spagnuolo, Angela Wahl, J. Victor Garcia, Susana T. Valente

**Affiliations:** 1 International Center for the Advancement of Translational Science, University of North Carolina at Chapel Hill, Chapel Hill, NC 27599, USA; 2 Center for AIDS Research, University of North Carolina at Chapel Hill, Chapel Hill, NC 27599, USA; 3 Division of Infectious Diseases, Department of Medicine, University of North Carolina at Chapel Hill, Chapel Hill, NC 27599, USA; 4 Department of Immunology and Microbiology, University of Florida Scripps Biomedical Research, Jupiter, FL 33458, USA

**Keywords:** HIV-1, Tat inhibitor, resistance, latency promoting agent, transcription, dCA

## Abstract

HIV resistance to the Tat inhibitor didehydro-cortistatin A (dCA) in vitro correlates with higher levels of Tat-independent viral transcription and a seeming inability to enter latency, which rendered resistant isolates more susceptible to CTL-mediated immune clearance. Here, we investigated the ability of dCA-resistant viruses to replicate in vivo using a humanized mouse model of HIV infection. Animals were infected with WT or two dCA-resistant HIV-1 isolates in the absence of dCA and followed for 5 weeks. dCA-resistant viruses exhibited lower replication rates compared to WT. Viral replication was suppressed early after infection, with viral emergence at later time points. Multiplex analysis of cytokine and chemokines from plasma samples early after infection revealed no differences in expression levels between groups, suggesting that dCA-resistance viruses did not elicit potent innate immune responses capable of blocking the establishment of infection. Viral single genome sequencing results from plasma samples collected at euthanasia revealed that at least half of the total number of mutations in the LTR region of the HIV genome considered essential for dCA evasion reverted to WT. These results suggest that dCA-resistant viruses identified in vitro suffer a fitness cost in vivo, with mutations in LTR and Nef pressured to revert to wild type.

## 1. Introduction

Our group discovered and characterized the potent HIV-1 Tat inhibitor, didehydro-Cortistatin A [1]. dCA inhibits Tat-dependent HIV transcription by binding to the Tat basic domain, blocking the Tat-TAR feedback loop that drives HIV-1 transcription to exponential levels [2]. dCA has the unique property of driving HIV-1 gene expression into a state of persistent latency, refractory to viral reactivation by different latency-reversing agents (LRAs), as shown in cell lines and in primary CD4+ T cells isolated from antiretroviral therapy (ART)-suppressed people living with HIV-1 (PLWH) [3,4]. Importantly, when dCA was added to ART-suppressed humanized mice, levels of HIV-1 mRNA were systemically reduced in tissues and delayed viral rebound upon treatment interruption [3].

**Figure 1:**
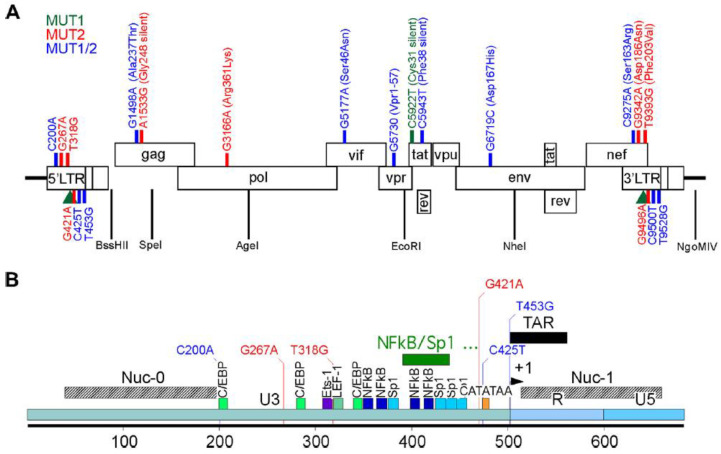
Schematic representation of HIV NL4.3 and the MUT1 and MUT 2 mutations acquired to become resistant to dCA in vitro. (**A**) Mutations found across the genome. (**B**) Details of the mutations present in the HIV-1 5′ LTR. In green, mutations found in MUT1 dCA-resistant HIV-1 variant; in blue, mutations found in MUT2 dCA-resistant HIV-1 variant and in red, mutations found in both MUT1 and MUT2 dCA-resistant HIV-1 variants. The green rectangle depicts a 49-nucleotide insertion found in a MUT1 variant. Amino acid changes are indicated in parenthesis. Previously published in Mousseau et al., mBio 2019 [5].

Foreseeing the addition of latency-promoting agents (LPAs) such as dCA to the ART regimen in the clinic, and to study the molecular mechanism of drug activity, we investigated the genetic barrier of HIV-1 to resistance to dCA [5]. The HIV-1 NL4-3 strain was passaged for 12 months in naïve HeLa-CD4 cells in the presence of increasing doses of dCA. Two dCA-resistant HIV-1 variants were isolated, MUT1 and MUT2, capable of replicating in 1 µM of dCA (~1000 times the EC_50_ of dCA), while remaining sensitive to other antiretroviral (ARVs) drugs. Next-generation sequencing of the two dCA-resistant variants revealed 9 point mutations and two NF-κB/one Sp1 site insertion in MUT1, and 13 point mutations in MUT2, throughout the LTR, Gag, Pol, Vif, Vpr, Tat, Env and Nef (Figure 1). A detailed work of reverse genetics identified mutations in HIV-1 LTR, Nef and Vpr region as fundamental for resistance to dCA. Mutations in both MUT1 and MUT2 Tat proteins were silent, and no mutations were detected in TAR. Mutations in Tat and TAR are known to limit viral replication [6,7,8] To evade a Tat inhibitor such as dCA, it was somewhat expected that we observe variants that acquired greater Tat-independent transcription fitness to augment Tat production to quench dCA. The nucleotide changes in the LTR promoted a 10–20-fold increase in basal transcription, and Nef mutations and a truncation of Vpr resulted in the upregulation of NF-κB activity. This higher transcriptional fitness resulted in higher viral production and consequently higher cytopathic effects on the host cell, as well as increased cytotoxic T lymphocyte (CTL)-mediated killing [5].

To our knowledge, these were the first described Tat-inhibitor-resistant HIV-1 isolates. Tat-independent viral replication is often associated with low transcriptional rates [6,7,8,9] since Tat is the main regulator of transcription. However, these identified HIV-1 variants resistant to dCA are Tat-independent, but transcriptionally very strong. These novel HIV-1 variants provide a unique opportunity to further the understanding of the role of Tat in HIV-1 transcription and establishment of latency, specifically because Tat is still expressed in these viruses. By being transcriptionally strong Tat-independent viruses, these isolates could help determine whether the establishment of latency is regulated by the Tat-TAR feedback loop or solely the result of cellular quiescence, as is still an open question in the field [10,11,12,13,14]. Here, we evaluated the replication fitness and kinetics of dCA-resistant HIV-1 in vivo, using a humanized mouse model of HIV-1 infection.

## 2. Materials and Methods

### 2.1. Construction of Humanized Mice

Humanized mice were prepared as previously described [15,16,17,18]. Briefly, an approximately 2-mm piece of human liver tissue was sandwiched between two pieces of autologous thymus tissue (Advanced Bioscience Resources) under the kidney capsule of sublethally irradiated (300 cGy) 6 to 8-week-old NOD.Cg-*Prkdc^scid^ Il2rg^tm1Wjl^*/SzJ (NSG, The Jackson Laboratory) mice. Following implantation, mice were transplanted intravenously with hematopoietic CD34+ stem cells isolated from autologous human liver tissue. Human immune cell reconstitution was monitored in the peripheral blood of humanized mice by flow cytometry every 3 to 4 weeks. Mouse experiments were conducted in accordance with NIH guidelines for the housing and care of laboratory animals and in accordance with protocols reviewed and approved by the Institutional Animal Care and Use Committee (IACUC) at the University of North Carolina, Chapel Hill.

In the current study, a total of 26 mice were used (males and females). The age range at humanization was 12 to 16 weeks old. All animals were successfully humanized, as measured by the frequency of human (h) CD45+ cells, CD3+ cells of hCD45+ cells, CD4+ cells of hCD45+ cells, and CD8+ cells of hCD45+ cells (Supplementary Table S1).

### 2.2. Production of Viral Stocks for Infection

Stocks of HIV-1_NL4.3_ (WT), HIV-1_NL4.3-MUT1_ (MUT1), and HIV-1_NL4.3-MUT2_ (MUT2) were prepared by transfecting HEK293T cells with pNL4.3 (NIH AIDS Reagent Program), pET28b-NL43-MUT1 (synthesized by ACGT Inc.) and pET28b-NL43-MUT2 (synthesized by ACGT Inc., Germantown, MD, USA) using *Trans*IT^®^-LT1 Transfection Reagent (Mirus), following the manufacturer’s protocol. Viral supernatant was collected 72 h post-transfection and filtered through a 0.45 µm filter. Viruses were titrated (TCID_50_) by infecting TZM-bl cells (NIH AIDS Reagent Program) at multiple dilutions and incubated for 48 h. After the incubation period, cells were washed with PBS and lysed with Passive Lyses Buffer (Promega, Madison, WI, USA), followed by the addition of Luciferase Assay Reagent (Promega). Light production was measured at 560 nm absorbance. The TCID_50_ was calculated according to the Reed and Muench method, as previously described (Reed and Muench 1938). Wells with RLU < 2.5 times background were considered negative for the calculation. Each viral stock titer was performed in triplicate, and at least two different titer determinations were performed for each batch of virus.

### 2.3. Plasma HIV-1 Viral Load Quantification

Plasma viral load was measured longitudinally by RT–qPCR using a TaqMan RNA to-CT 1-step kit (Applied Biosystems, Foster City, CA, USA ) as described previously [19]. The sequences for forward and reverse primers and the TaqMan probe are 5′-CATGTTTTCAGCATTATCAGAAGGA-3′, 5′-TGCTTGATGTCCCCCCACT-3′, and 5′-FAM-CCACCCCACAAGATTTAAACACCAT-GCTAA-Q-3′, respectively. The real-time PCR was carried out in 96-well plates using the ABI 7500 Fast Real Time PCR System (Applied Biosystems).

### 2.4. Cell-Associated HIV-1 RNA and DNA

RNA was extracted using QIAamp viral RNA Mini Kit (Qiagen, Hilden, Germany) according to the manufacturer’s protocol including the optional RNase-free DNase step and analyzed using one-step reverse-transcriptase qPCR (ABI custom TaqMan Assays-by-Design) [20]. All samples were run and analyzed on an ABI 7500 Fast Real-Time PCR System (Applied Biosystems). The levels of proviral DNA in tissues and peripheral blood were quantified by RT–qPCR analysis, as previously described [21,22]. Human gamma globin DNA was amplified in parallel to detect the presence of human cells.

### 2.5. Mononuclear Cell Isolation

Mononuclear cells (MNCs) were isolated from tissues, as previously described [21,23]. Briefly, mononuclear cells from the human thymic organoid, lymph nodes, and spleen were isolated by passing tissue through a cell strainer. Bone marrow MNCs were collected by homogenizing bones with a mortar and pestle and passing through a cell strainer. The lung and liver were cut into small pieces, digested with collagenase/DNase solution, then passed through a cell strainer. MNCs were then purified by Percoll density gradient centrifugation. Tissue red blood cells were lysed with ACK lysis buffer.

### 2.6. Immunophenotyping by Flow Cytometry

Immunophenotyping was performed on peripheral blood samples collected longitudinally and at the study endpoint on whole blood and on mononuclear cells isolated from the tissues of humanized mice. Prior to antibody incubation, Ig-binding sites were blocked. The fluorochrome-conjugated antibodies included CD45-V500 (HI30, BD Biosciences, San Jose, USA), CD3-APC-R700 (UCHT1, BD Biosciences, San Jose, USA), CD4-APC-H7 (RPA-T4, BD Biosciences, San Jose, USA), CD8-FITC (SK1, BD Biosciences, San Jose, USA), CD19-PE-Cy7 (SJ25C1, BD Biosciences, San Jose, USA), CD27-PE (M-T271, BD Biosciences, San Jose, USA), CD38-APC (HB7, BD Biosciences, San Jose, USA), CD45RA-Pacific Blue (F8-11-13, Bio-Rad, Hercules, USA), HLA-DR-PerCP (L243, BD Biosciences, San Jose, USA), mouse IgG1k-APC (MOPC-21, BD Biosciences, San Jose, USA), mouse IgG1k- Pacific Blue (MOPC-21, BD Biosciences, San Jose, USA), mouse IgG1k-PE (MOPC-21, BD Biosciences, San Jose, USA) and mouse IgG2ak-PerCP (X39, BD Biosciences, San Jose, USA). After antibody incubation, blood samples were lysed with t 1× BD FACS lysing solution (BD Biosciences, San Jose, USA). Samples were then analyzed on a BD LSRFortessa instrument (BD Biosciences, San Jose, USA).

### 2.7. Plasma Cytokine and Chemokine Analysis

Plasma levels of human IFNγ and IFNα2 mice were determined using undiluted samples using a Milliplex MAP kit (Millipore, HCYTMAG-60K-PX41, Burlington, MA, USA) on a Luminex MAGPIX instrument by the University of North Carolina, Chapel Hill Center for AIDS Research Virology Core Laboratory.

### 2.8. Single Genome Sequencing Assay

Plasma viral RNA was submitted to cDNA synthesis using the SuperScript™ III First-Strand Synthesis System kit (Invitrogen, Waltham, MA, USA), according to the manufacturer’s protocol with a few alterations. Briefly, 2.5 µL of deoxynucleoside triphosphates (dNTPs) (10 mM) and 2.5 µL of HIV-specific primer (9603 5′-TAAAGCTTGCCTTGAGTGCTCAAA; 10 µM) were added to 20 µL of viral RNA, followed by denaturation at 65 °C for 5 min. Next, 25 µL of reverse transcription mix (5 mM MgCl_2_, 40 U RNase OUT, dithiothreitol 1mM, 1× RT Buffer, and 200 U SuperScript III Reverse Transcriptase) was added to the denatured viral RNA (V_final_ = 50 µL). The reaction was incubated at 50 °C for 50 min, and then at 85 °C for 10 min.

To obtain PCR products derived from single cDNA molecules, viral cDNA was serially diluted 1:3 in 5 mM of Tris-HCl (pH 8.0) to a maximum dilution of 1:6561. Ten separate nested-PCR amplifications were performed for each cDNA dilution as follows: 2 µL of diluted cDNA was added to 8 µL of PCR mix (V_final_ = 10 µL), containing 1× DreamTaq™ Hot Start PCR Master Mix (Thermo Scientific) and 200 nM of each primer (4630 forward 5′-GGATCAAGCAGGAATTTGGC and 9603 reverse 5′-TAAAGCTTGCCTTGAGTGCTCAAA). The 4973 bp amplicon was submitted to a nested-PCR using the same protocol with the following primers: 9129 forward (5′-ACCACACACAAGGCTACTTC) and 9563 reverse (5′-GCTCTCTGGCTAACTAGGGAAC). The 456 bp nested-PCR amplicon was identified by agarose gel electrophoresis. According to Poisson’s distribution, the cDNA dilution yielding positive amplicons in 3 out of 10 nested PCRs contains one copy of cDNA per positive nested PCR.

Once the endpoint dilution was determined for the mouse plasma sample, 2 µL of diluted cDNA was added to 8 µL of PCR mix (V_final_ = 10 µL), containing 1x DreamTaq™ Hot Start PCR Master Mix (Thermo Scientific, Waltham, MA, USA) and 200 nM of each primer (4630 forward and 9603 reverse) in a 96-well plate format. The 4973 bp product of the first PCR encompasses the HIV-1 genome regions containing mutations necessary for complete dCA evasion (Vpr, Env, Nef, and 3′ LTR). This PCR product was used as a template for three independent nested PCRs for Vpr (2302 forward 5′-AAGCCACCTTTGCCTAGTG and VprB reverse 5′-CTCCGCTTCTTCCTGCCAT), Env (F1 forward 5′-AGCGGGAGAATGATAATGGAGA and R2 reverse 5′-CACAGGCCTGTCCAAAGGTA), and Nef/3′LTR (9129 forward and 9563 reverse). Positive wells (i.e., single clones) were identified by agarose gel electrophoresis and sequenced by direct dideoxyterminator (performed by Eurofins Genomics, Ebersberg, Germany) in both directions using overlapping internal primers. Sequences from each genomic region were aligned and compared to the NL4.3 consensus sequence by using Geneious Prime^®^ software (v. 2022.2.2). Sequencing reactions that identified more than one amplified genome were not included in the analyses.

### 2.9. Statistical Analysis

**Figure 2:**
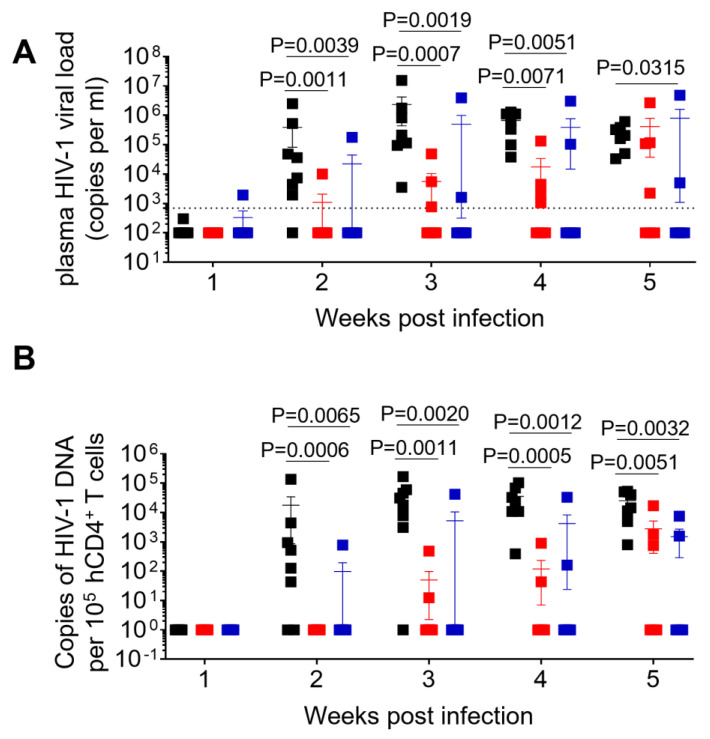

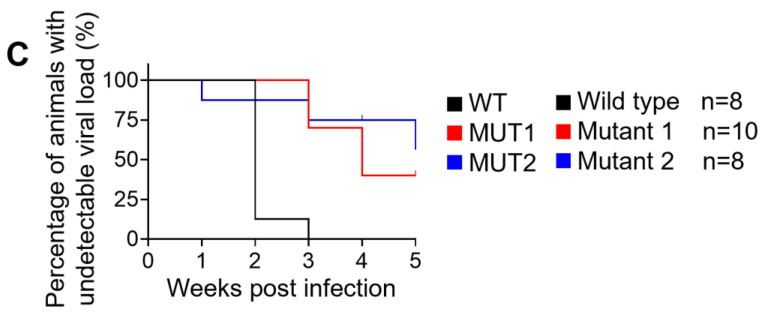
Longitudinal analysis of plasma viral load and cell-associated HIV-1 DNA. (**A**) Plasma HIV-1 viral load was monitored longitudinally by real-time PCR. (**B**) Levels of cell-associated HIV-1 DNA were monitored longitudinally by real-time PCR. (**C**) Kaplan–Meier curve depicts the percent of animals with undetectable plasma viral load following exposure. Data in (**A**,**B**) are expressed as mean± SEM. Statistical significance was determined using a two-sided Kruskal–Wallis test in (**A**,**B**), and a Mantel–Cox test in (**C**). *p* values between WT and MUT1, WT and MUT2 in (**C**) are <0.0001 and 0.0033, respectively.

Statistical analyses were performed using GraphPad Prism Software (v. 8). Statistical significance was considered when *p* < 0.05. A two-sided Kruskal–Wallis test was used to assess the statistical significance of the differences between groups for all analyzed parameters, except the Kaplan–Meier curve (Figure 2C) where the Mantel–Cox test was used. No statistical methods were used to predetermine the sample size. Investigators were not blinded to group allocations or when assessing outcomes.

## 3. Results

### 3.1. Replication Kinetics of dCA-Resistant HIV-1 Variants in Humanized Mice

**Figure 3:**
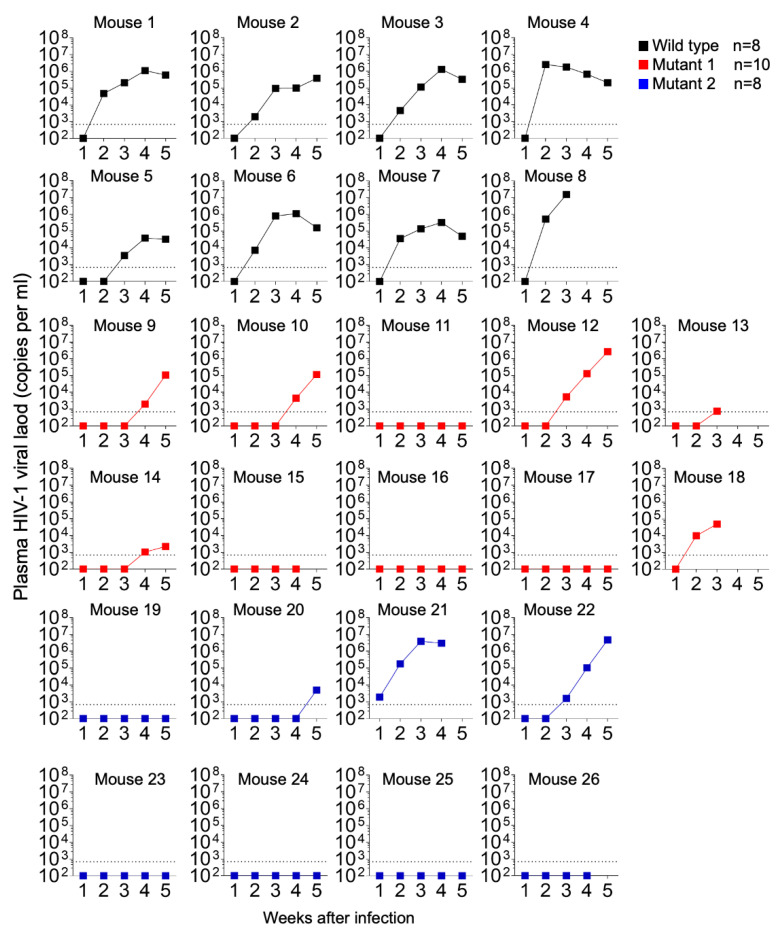
HIV-1 plasma viral load monitored longitudinally by qPCR.

A total of 26 humanized mice were intravenously infected with 30,000 TCID_50_ of wild-type NL4.3 (WT) (n = 8), dCA-resistant NL4.3 variant 1 (MUT1) (n = 10) and dCA-resistant NL4.3 variant 2 (MUT2) (n = 8). Animals were followed for 5 weeks and whole blood was collected longitudinally to assess infection markers. Contrary to our expectation, HIV-1 plasma viral load determined by RT-qPCR revealed that WT-infected mice sustained significantly higher systemic replication levels throughout weeks 2 and 5 post-infection (Figure 2A and Figure 3). Similarly, HIV-1 proviral DNA levels per total human CD4+ T cells quantified by qPCR revealed similar results, with WT-infected mice harboring significantly higher levels of proviral DNA compared to MUT1- and MUT2-infected animals (Figure 2B and Figure S1). The Kaplan–Meier plot in Figure 2C depicts the percentage of mice with undetectable plasma viral load as a function of time postexposure. The plot shows statistical significance between the percentage of WT-infected animals that became HIV-positive and the percentage of MUT1- and MUT2-infected animals. These results reveal that MUT1- and MUT-2 infected mice presented a delay in the establishment of infection compared to WT-infected animals that had detectable plasma viral load by week 3 postexposure. Overall, 6 out of 10 (60%) and 5 out of 8 (62.5%) animals infected with MUT1 and MUT2, respectively, had no detectable plasma viremia 5 weeks postexposure.

**Figure 4:**
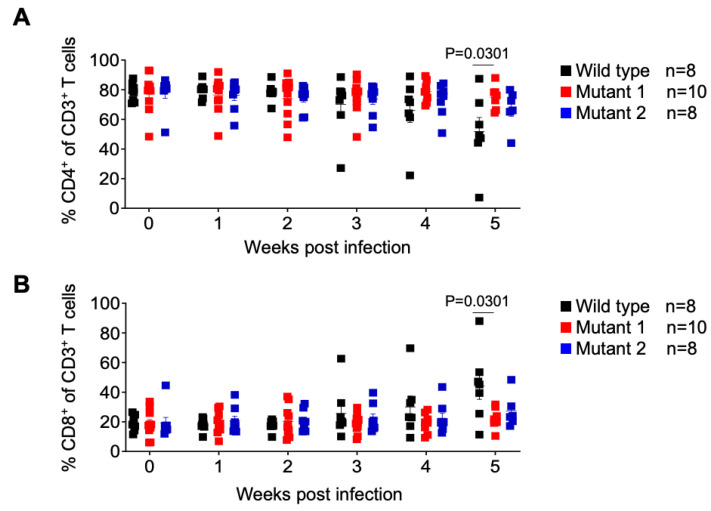
Analysis of human T cell levels in peripheral blood of HIV-1 infected humanized mice. Frequency of human CD4+ (**A**) and CD8+ (**B**) T cells in the peripheral blood was monitored longitudinally by flow cytometry. Data are expressed as mean ± SEM. Statistical significance was calculated using a two-sided Kruskal–Wallis test.

The frequency of CD4+ T cells (CD4+/CD3+ of total human CD45+) and CD8+ T cells (CD8+/CD3+ of total human CD45+) were assessed longitudinally. No significant differences between animal groups were observed; except at week 5, when WT-infected animals showed a significant decrease in the mean frequency of CD4+ T cells and a mean increase in CD8+ T cells (Figure 4). These results show that infection with the WT virus resulted in the depletion of CD4+ T cells mirroring the natural history of HIV infection in humans.

**Figure 5:**
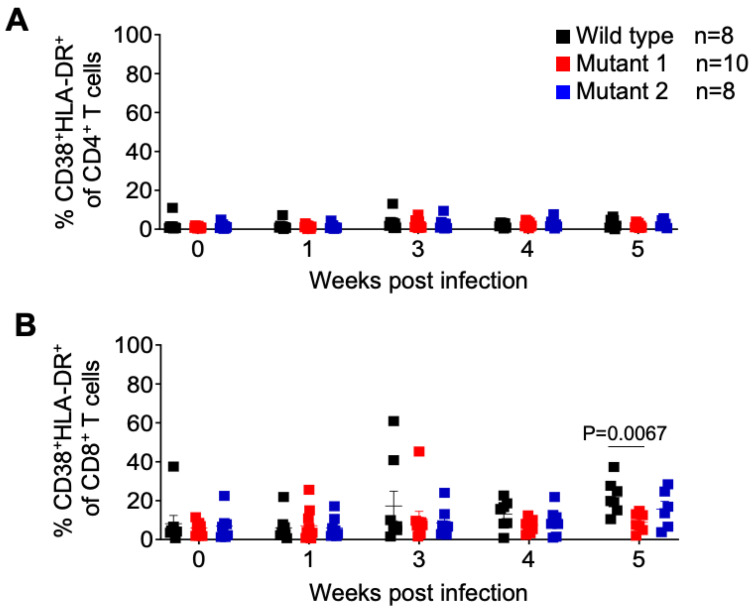
Frequency of activated T cells in peripheral blood of HIV-1 infected humanized mice. Activated CD4+ (**A**) and CD8+ (**B**) T cells in peripheral blood were identified by flow cytometry using anti-human CD38 and HLA-DR antibodies. Shown is a longitudinal analysis following HIV-1 exposure. Data are expressed as mean ± SEM. Statistical significance was calculated using a two-sided Kruskal–Wallis test.

We also monitored the levels of activated CD4 and CD8 T cells throughout the course of the infection. No differences in the frequency of activated CD4+ T cells (CD38+/HLA-DR+ of total CD4+ T cells) and activated CD8+ T cells (CD38+/HLA-DR+ of total CD8+ T cells) throughout the experiment, except at the endpoint (week 5), when a significantly higher mean of activated CD8+ T cells was observed in WT-infected animals (Figure 5). 

Together these data suggest that dCA-resistant HIV-1 variants are not fit to replicate in vivo, do not induce CD4 T cell depletion, and do not induce T cell activation. These results are contrary to our expectations based on previous in vitro results showing better in vitro fitness for the dCA-resistant variants [5]. 

### 3.2. dCA-Resistant HIV-1 Variants Establish Systemic Infection in Humanized Mice

**Figure 6:**
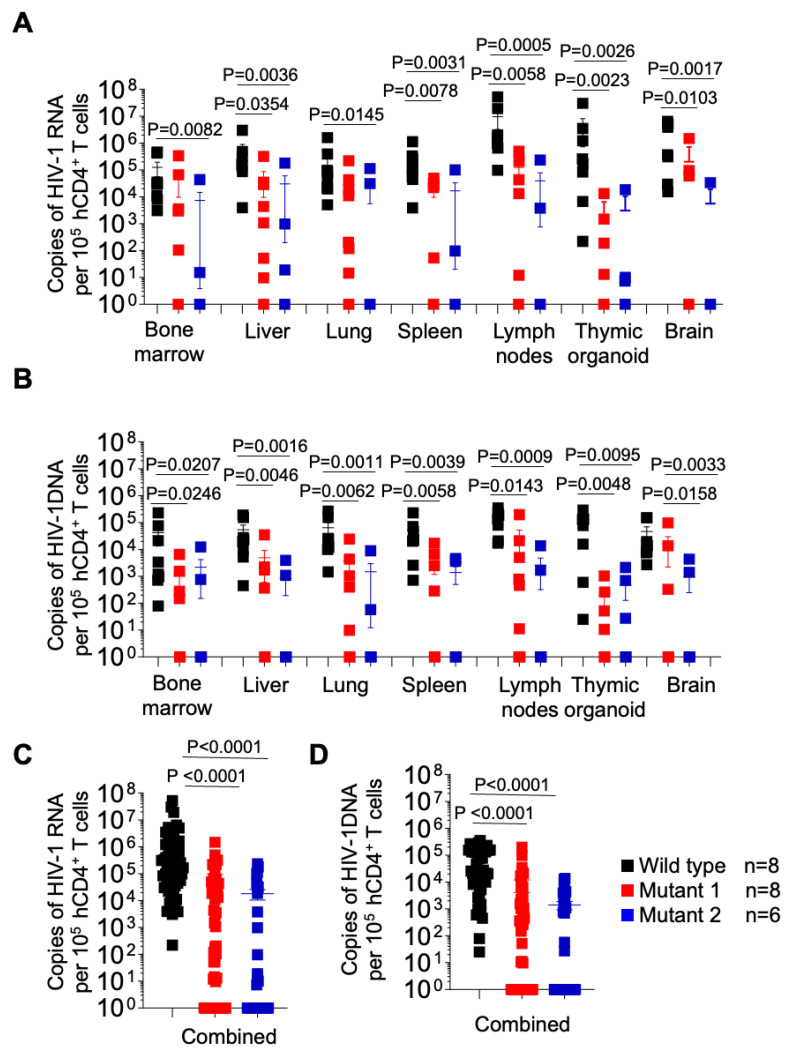
Longitudinal analysis of cell associated HIV-1 RNA and proviral DNA in tissues from HIV-1 infected humanized mice. Levels of cell-associated RNA (**A**) and proviral DNA (**B**) per 10^5^ human CD4+ T cells in tissues of HIV-1-infected humanized mice were determined at necropsy. (**C**,**D**) depict the levels of cell-associated RNA and proviral DNA for all tissues combined, respectively. Data are expressed as mean ± SEM. Statistical significance was calculated using a two-sided Kruskal–Wallis test.

After 5 weeks of infection, tissues were collected for analysis. Cell-associated viral RNA (CA-vRNA) and proviral DNA in the bone marrow, liver, lung, spleen, lymph nodes, thymic organoid and brain were quantified by qPCR and expressed as viral copies per 10^5^ human CD4+ T cells (Figure 6). HIV-1 RNA was found in all tissues from WT-infected animals and in all but one of the animals infected with MUT1 and in all but one animal infected with MUT2. Importantly, significantly lower levels of HIV RNA were found in all tissues analyzed from the animals infected with either MUT1 or MUT2. (Figure 2A). These results indicate significantly reduced levels of systemic HIV-1 replication of both variant viruses (Figure 6A). Consistent with the levels of cell-associated RNA present in tissues, proviral DNA was detected in all tissues from the animals infected with the WT virus and all but one mouse infected with MUT1, and one mouse infected with MUT2 (Figure 6B). Proviral DNA levels were significantly lower in animals infected with MUT1 and MUT2 variants. However, no statistical differences were observed in the levels of CA-vRNA and proviral DNA between MUT1- and MUT2-infected animals.

**Figure 7:**
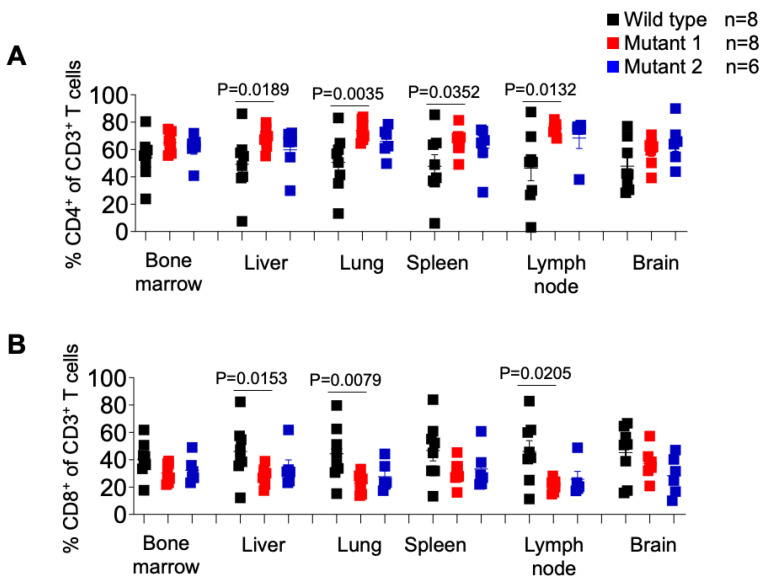
Frequency of CD4+ or CD8+ T cells in tissues of HIV-1-infected humanized mice. Percentage of human CD4+ (**A**) and CD8+ (**B**) T cells in tissues of HIV-1-infected humanized mice was determined by flow cytometry. Data are expressed as mean ± SEM. Statistical significance was calculated using a two-sided Kruskal–Wallis test.

We also analyzed the levels of human CD4+ T cells in tissues of WT-, MUT1-, and MUT2-infected mice and observed a similar pattern observed as in the periphery (Figure 7A). The levels of human CD4+ T cells in the liver, lung, spleen, and lymph node were lower in WT-infected mice compared to MUT-1- and MUT-2-infected mice. However, the differences observed between WT and MUT2-infected animals did not reach statistical significance. We did not observe differences in the levels of CD4+ T cells in the bone marrow and in the brain between animal groups. 

With respect to the frequency of CD8+ T cells in different tissues of HIV-1-infected animals (Figure 7B), we observed a significantly higher percentage of these cells in the liver, lung, and lymph node in WT-infected mice compared to MUT1-infected mice. No differences were observed between WT- and MUT2-infected animals. The frequency of CD8+ T cells did not differ significantly in the bone marrow, spleen, and brain tissues (Figure 7B).

**Figure 8:**
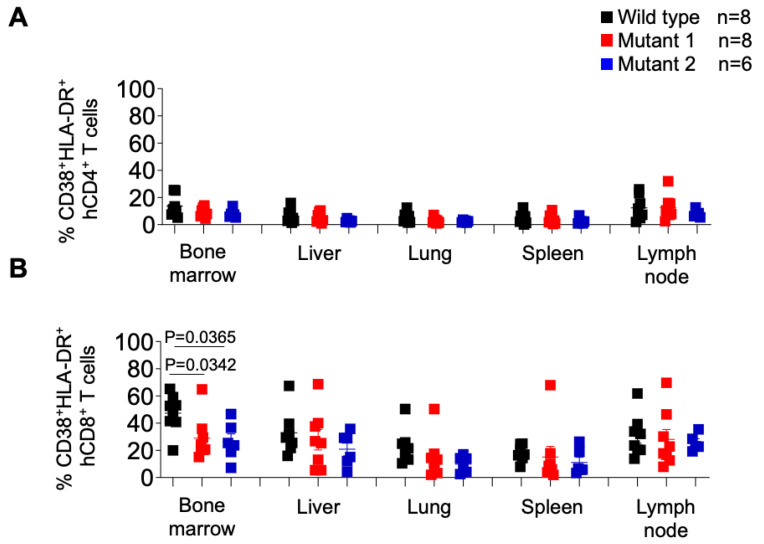
Frequency of activated CD4+ and CD8+ T cells in tissues of HIV-1-infected humanized mice. Activated human CD4+ (**A**) and CD8+ (**B**) T cells in tissues of HIV-1-infected humanized mice were identified by the co-expression of hCD38 and HLA-DR. Data are expressed as mean ± SEM. Statistical significance was calculated using a two-sided Kruskal–Wallis test.

Intriguingly, when the levels of CD8+ T cell activation were characterized (Figure 8B), we observed a significantly higher percentage of activated CD8+ T cells in the bone marrow of WT-infected mice, and no other tissue, compared to both MUT1- and MUT2-infected animals. No statistically significant differences were observed in the levels of CD4+ T cell activation.

Collectively these results suggest that dCA-resistant HIV-1 variants were significantly less fit to replicate in vivo when compared to the WT virus. 

### 3.3. dCA-Resistant HIV-1 Variants Do Not Elicit IFN Responses

**Figure 9:**
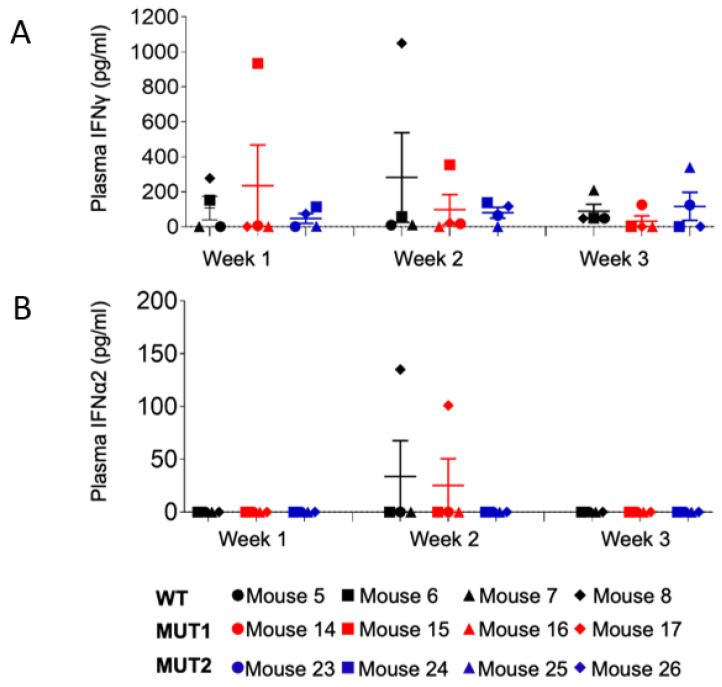
Levels of human IFNγ and IFNα in the plasma of HIV-1 infected humanized mice. Levels of plasma IFNγ (**A**) and plasma IFNα2 (**B**) in HIV-1-infected humanized mice at weeks 1, 2, and 3 post exposure were determined using a bead array kit. Data are expressed as mean ± SEM. Statistical significance was calculated using a two-sided Kruskal–Wallis test.

Plasma levels of IFN-γ and IFN-α2 were evaluated for 12 animals (4 per group) at weeks 1, 2, and 3 postexposure (Figure 9), since these responses elicit broadly anti-viral effects. IFN-α2 levels were barely detected in plasma at the time points analyzed, with the exception of mice 8 (WT-exposed) and 17 (MUT1-exposed). At week 3 post-exposure, mouse 8 presented the highest plasma viral load (10^7^ HIV-1 copies/mL, Figure 3) among all analyzed animals, and deceased by week 4 post-exposure, indicating that the levels of IFN responses were possibly due to high levels of viral replication (Figure 3 and Figure 9). Interestingly, this animal also presented high levels of IFN-γ at week 2 post-exposure. Regarding mouse 17, plasma viral load was not detected throughout the 5-week experiment. Thus, we cannot suggest a correlation with IFN responses since animals 15 and 16, among the ones exposed to the MUT1 variant, presented the same HIV-1 viral load pattern as animal 17 with no detectable levels of IFN-α2 (Figure 3 and Figure 9).

### 3.4. Ability of dCA-Resistant HIV-1 Variants to Establish Infection Correlates with Reversion to Wild-Type HIV-1

HIV-1 variants resistant to dCA (1 nM EC_50_) were isolated by passaging HIV-1 NL4.3, every week, onto naive HeLa-CD4 cells in the presence of increasing concentrations of dCA (0.1 nM to 1 µM) over a period of 12 months. Viral fitness and resistance to dCA were confirmed in different T cell lines (CEM-SS and Jurkat) and in primary CD4+ T cells [5]. Thus, it was unexpected to observe lower viral replication rates of dCA-resistant HIV-1 variants in vivo, and most animals exposed to these variants did not even have an established infection. We thus hypothesize that the dCA-resistant HIV-1 variants selected in vitro in HeLa-CD4 cells lose fitness in vivo and that the few animals in which a delayed infection was observed, did so by reverting to the WT virus. As such, endpoint plasma samples from humanized mice infected with WT, MUT1, and MUT2 were submitted to single genome sequencing of the four HIV-1 genomic regions containing mutations essential for dCA evasion and fitness (5′ LTR, Vpr, Env, and Nef).

The 5′ LTR of the HIV-1 genome serves as the transcriptional promoter and, thus, one might expect this region to be subjected to intense selective pressure when resistant to a transcriptional inhibition, such as dCA. Indeed, we previously showed that the HIV-1 LTR carries the most mutations associated with dCA resistance, including a 49-nucleotide insertion containing two additional NF-kB binding sites [24]. In vivo, we observed the C200A mutation, located at the 3′ extremity of the nucleosome 0 binding site [25,26], only reverted to WT in one clone of animal 10 of a total of 69 single cDNA clones analyzed for dCA-resistant-HIV-1-infected mice (Table 1). However, the mutation G267A present only in the MUT2 variant, reverted to WT in 100% of the clones analyzed. We did not find any apparent relevance for this 5′ LTR mutation in the literature, but we found some relevance in the context of nef (discussed below). The MUT2 dCA-resistant variant harbors two extra mutations not found in the MUT1 variant. The T318G mutation is located in the first nucleotide of the TCF-4/LEF-1 binding site, where the TCF-4/β-catenin/SMAR1 complex is known to repress HIV-1 transcription [27,28]. This mutation reverted to WT in only one out of the seven (8%) clones analyzed for animal 22 and in none of the four clones analyzed for animal 21. On the other hand, the mutation G421A, located just upstream of the TATA box, showed reversion in 100% of the clones in animal F634 and in a minority of the clones in animal F182 (28%). The mutation C425T, located at position 1T of the TATA box, was found in both MUT1 and MUT2 variants. This mutation changes the CATATAA box of subtype B HIV-1 into TATATAA, which seems to increase viral fitness [29]. Only a few MUT1-infected mice (F627, F631, and F686) analyzed reverted to WT at C425T, while the majority of the clones from MUT2-infected mice (F182 and F634) reverted to WT at C425T (Table 1). The last mutation in the 5′ LTR of dCA-resistant HIV-1 variants is T453G, located at position -2 of the transcription start site (TSS) and at the basis of the TAR hairpin. This mutation reverted to WT in all MUT1-infected mice (9, 10, and 12) and in animal 21, infected with the MUT2 variant. Moreover, MUT2-infected animal 22 presented the T453G reversion in 14% of the single cDNA clones analyzed (Table 1).

Both dCA-resistant HIV-1 variants carry a deletion in vpr (G5730) that translates into a truncated form of Vpr (Vpr_1–57_). In vitro, the presence of Vpr_1–57_ resulted in a two-fold increase in NF-κB-driven transcription [5]. Here, we found no reversions to the WT in dCA-resistant-HIV-1-infected mice (Table 1). In env, MUT1 and MUT2 carried a mutation (G6719C) leading to an amino acid change (Asp167His) in the V2 loop of gp120, that though was not necessary for total dCA evasion, increased viral fitness in vitro [5]. In vivo, this mutation persisted in both MUT1- and MUT2-infected mice; however, animal 21 acquired another mutation (G6712A) between loops V1 and V2 that leads to an amino acid change (Ser164Asn). This mutation has been associated in vitro with viral adaptation to replicate in CCR5 low cell lines [30]. Here, we extrapolate that this mutation may be correlated with the ability of MUT2 to establish and sustain infection in animal 21 to levels similar to WT-infected animals, as opposed to MUT1-infected animals that did not acquire this sequence. Unfortunately, we were not able to sequence single env clones for the other MUT2-infection that presented detectable viral load to confirm our hypothesis (animals 20 and 22) (Table 1, Figure 2A and Figure 3).

Three mutations in nef were also associated with dCA evasion [5]. MUT1 and MUT2 variants both carried the C9275A mutation, located in a Nef residue known to interact with AP-2 in T cells to form a complex and downregulate CD4 [31]. In vivo, we observed that this mutation did not revert to WT in any of the 62 single cDNA clones sequenced (Table 1). The MUT2 variant also has a G9342A mutation. This mutation reverted to wild type in 100% of the clones sequenced from animals 21 and 22 infected with MUT2 (Table 1). Interestingly, this mutation is in a Nef residue that mimics the β-catenin binding site. The interaction between Nef and β-catenin has been confirmed in vitro and transfection of Nef in HEK293 cells specifically inhibits the Wnt pathway [32]. Lastly, the T9393G mutation present in MUT2 only reverted in one clone out of seven (8%) from animal 22 and no reversions were detected in any clone from animal 21 (Table 1). This mutation leads to an amino acid change (Phe203Val) in the C-terminal loop of Nef known to be involved in CD4 endocytosis [33].

Our plasma cDNA single genome sequencing data show that mutations in the 5′ LTR and in nef that confer resistance to dCA in vitro are under greater selective pressure to revert to WT. We hypothesize that dCA resistance mutations selected in vitro are not fit in vivo and revert to WT in the absence of dCA. Of note, it is possible that this would not be the case if animals were exposed to dCA-resistant HIV-1 variants in the presence of the drug; however, adding dCA treatment in the experimental design would make it difficult to control for the establishment of infection and replication rates since the WT virus would be hindered by dCA. The current experiment in the absence of dCA allowed us to compare replication fitness between WT and dCA-resistant HIV-1 in vivo.

## 4. Discussion

In this study, we explored the in vivo significance of HIV-1 variants resistant to the Tat inhibitor didehydro-Cortistatin A. Contrary to our in vitro findings showing that these isolates had higher transcriptional fitness which resulted in higher cytopathic effects, dCA-resistant HIV-1 variants in humanized mice either did not replicate or showed delayed replication. All humanized mice infected with the WT virus efficiently established a productive infection. Most mice exposed to dCA-resistant HIV-1 variants did not establish infection. A few of the animals exposed to MUT1 and MUT2 dCA-resistant variants established infection late after exposure (week 3), presenting detectable viral load and peak replication similar to WT-infected animals by 5 weeks post-infection. The lack of viral replication could not be attributed to higher IFN responses. Rather, genomic analysis of plasma viral RNA revealed reversion to wild type in at least half of the mutations carried by these variants responsible for dCA evasion. These results suggest that the dCA-resistant HIV-1 variants have a fitness cost in vivo and are pressured to WT reversion in order to establish infection.

Tat is the main regulator of HIV-1 transcription and Tat-independent viral replication is often associated with low transcriptional rates [9]. Our dCA-resistant HIV-1 variants, nonetheless, are Tat-independent while transcriptionally very strong in vitro. These novel viruses could provide us with a unique opportunity to further our knowledge of the role that Tat plays in HIV transcription and the establishment of latency. Latency has been extensively associated with the cell activation state (cell-state dependent latency model), in which viral latency is associated with the infected cell transitioning to a resting state, characterized by low levels of intracellular transcription factors, cofactors, and repressive chromatin modifications that block or limit viral transcription. However, this model does not explain, for instance, findings suggesting that latency reversal and HIV-1 transcription are not always directly coupled to cellular reactivation and availability of transcription factors [34,35,36]. An alternative model, dubbed the Tat-TAR dependent latency model, suggests that viral latency may be directly associated with the expression and overall function of Tat. Evidence to support this alternative model includes (1) the establishment of latency in activated primary CD4+ T cells without prior return to a resting state [10]; (2) the presence of latently infected cell lines with mutations in Tat or TAR [6,7]; (3) the presence of short, prematurely terminated RNA transcripts in latently infected cells from viremic asymptomatic individuals and aviremic patients on ART [37,38], indicating that transcription is being initiated in these latently infected cells and 4) the ability of exogenous Tat to rescue viral expression from latently infected cells [38,39,40]. Whether HIV-1 latency is the result of cell functional state at the moment of infection or is controlled by a viral intrinsic mechanism, or is the consequence of both, remains to be determined and these Tat-independent viruses are a unique tool for such studies.

The dCA-resistant variants evaluated here were isolated in vitro using HeLa-CD4 cells, as previously described [5]. These epithelial cells are not natural targets for HIV-1 infection; however, we confirmed resistance to dCA in primary CD4+ T cells. Nevertheless, one can still speculate whether we would have had a different outcome if dCA resistance were selected in a more biologically relevant cell line, such as a T cell line, or in vivo using humanized mice. Both approaches are currently being evaluated. The development of dCA resistance in vitro was a long process that took 12 months and required up to 13 nt changes [5], highlighting a high genetic barrier. For instance, among the six classes of ARVs, protease inhibitors (PIs) have the highest genetic barrier to resistance, needing a minimum of three to four mutations for high-level resistance to lopinavir/r and darunavir/r [41].

The majority of the mutations conferring resistance to dCA were located in the 5′ LTR region of HIV-1 and these were the most sensitive to WT reversion, suggesting their significance for viral fitness in vivo. Of note, dCA-resistant HIV-1 variants are still sensitive to other ARVs, such as raltegravir and saquinavir, in vitro [5]. Reversion to WT is, however, not atypical and has been described for other HIV-1 drugs [42]. Thus, it is also possible that given the nature of transcription, which is dependent on many host factors, resistance to transcriptional inhibitors may not be easily observed in vivo. HIV transcription has two phases: a first Tat-independent phase, triggered by host factors (e.g., NF-κB/TFIIH), followed by a second Tat-dependent amplification phase, triggered when Tat reaches a certain threshold and recruits pTEFb. If we were to speculate, the loss of MUT1 and MUT2 viral replication fitness in vivo with reversion to WT could suggest that two phases of viral transcriptional amplification are needed for the HIV life cycle, since we observed negative selection against single-phase viruses (Tat-independent, MUT1 and MUT2). Indeed, we have previously reported that dCA does not inhibit at all Tat transactivation of stably expressed MUT1 and MUT2 promoters driving Luciferase expression, nor the WT LTR driving Luciferase when cells are infected with the MUT1 or MUT2 variants, suggesting that MUT viruses have a single transcription phase (host factor dependent, high expression levels) not modulated by Tat.

As for Nef and Vpr, these are accessory genes exerting relevant roles in vivo but are known to be less relevant in vitro [43]. We found no reversions to WT in Vpr and only one reversion to WT was observed in Nef MUT 2 G9342A in 100% of the clones sequenced from animals 21 and 22. This Nef residue has been suggested to mediate Nef interaction with β-catenin in HEK293 cells to specifically inhibits the Wnt pathway [32]. Our results suggest that this in vitro function of Nef may be needed for viral survival in vivo and thus pressured to revert to WT.

In sum, here we studied the replication fitness of dCA-resistant HIV-1 variants in vivo and concluded that though these in vitro-selected variants have high replication fitness and elevated cytopathogenicity in vitro they are not fit in vivo. Together our studies suggest that dCA-resistant viruses identified in vitro suffer a large fitness cost in vivo, with mutations in the LTR and Nef regions the most pressured to revert to wild type.

## Figures and Tables

**Table 1:** Percentage of single cDNA clones from plasma that reverted to wild type HIV-1.

Group	Animal	5’ LTR								VPR		ENV		NEF			
		# Clones	C200A	G267A	T318G	Insertion	G421A	C425T	T453G	# Clones	G5730Δ	# Clones	G6719C	# Clones	C9275A	G9342A	T9393G
**WT**	**1**	11	100%	100%	100%		100%	100%	100%	17	100%	12	100%	11	100%	100%	100%
	**4**	27	100%	100%	100%		100%	100%	100%	5	100%	30	100%	27	100%	100%	100%
**MUT1**	**9**	17	0%			24%		0%	100%	20	0%	17	0%	17	0%		
	**10**	13	8%			100%		12%	100%	5	0%	4	0%	6	0%		
	**12**	28	0%			100%		4%	100%	16	0%	8	0%	28	0%		
**MUT2**	**20**	0	-	-	-		-		-	0	-	0	-	0	-	-	-
	**21**	4	0%	100%	0%		100%	100%	100%	4	0%	5	0%	4	0%	100%	0%
	**22**	7	0%	100%	8%		20%	86%	14%	6	0%	-	-	7	0%	100%	8%

Shaded areas indicate absence of mutation at the indicated position. Dashes indicate data not available.

## Data Availability

All the data is included in the manuscript.

## Supplementary Materials

The following supporting information can be downloaded at: https://www.mdpi.com/article/10.3390/v15040950/s1, Table S1. Levels of human hematopoietic cells and human T cells in peripheral blood of humanized mice prior to HIV-1 exposure; Figure S1. Individual mice HIV-1 proviral DNA monitored longitudinally by qPCR.
