# Supplementary material for: Loss of In Vivo Replication Fitness of HIV-1 Variants Resistant to the Tat Inhibitor, dCA
Source: Viruses. 2023 Apr 12;15(4):950. doi: 10.3390/v15040950 (PMC10146675; doi:10.3390/v15040950)
Supplement: Supplementary file 1 [file viruses-15-00950-s001.zip › viruses-2284388-SI.pdf]

Supplementary Materials

Table S1. Levels of human hematopoietic cells and human T cells in peripheral blood of humanized mice prior to HIV-1 exposure.

Group	Mouse ID	% hCD45+	% CD3+ of hCD45+	% CD4+ of CD3+	% CD8+ of CD3+
WT	1	77.7	82.0	81.3	17.0
	2	51.0	74.7	84.4	14.2
	3	78.9	64.7	78.3	19.1
	4	23.4	79.4	71.3	23.1
	5	59.8	54.8	87.7	11.5
	6	29.8	86.3	70.8	26.5
	7	61.3	42.6	79.5	18.1
	8	73.4	69.6	73.2	24.8
	Mean ±SEM	50.7 ±9.0	61.8 ±8.8	69.9 ±8.6	17.6 ±2.4
MUT1	9	61.1	60.8	83.5	14.3
	10	77.7	87.8	80.6	17.5
	11	47.0	80.4	82.3	16.0
	12	73.3	78.8	72.4	25.9
	13	37.7	87.9	48.4	33.7
	14	90.1	89.8	92.9	6.2
	15	24.9	79.1	79.3	18.1
	16	62.2	33.7	66.8	30.5
	17	58.9	37.3	79.5	18.3
	18	25.3	36.6	93.0	6.0
	Mean ±SEM	51.2 ±7.8	61.7 ±8.6	71.4 ±7.4	17.7 ±2.8
MUT2	19	65.1	80.1	83.1	14.6
	20	69.0	77.9	80.5	17.5
	21	63.1	78.1	79.3	17.5
	22	59.4	87.2	81.5	15.9
	23	73.6	82.8	86.5	11.9
	24	59.6	79.6	82.6	14.9
	25	31.8	25.6	51.2	44.6
	26	71.9	56.7	81.0	17.2
	Mean ±SEM	55.8 ±7.2	64.2 ±9.3	70.7 ±8.2	18.5 ±3.3

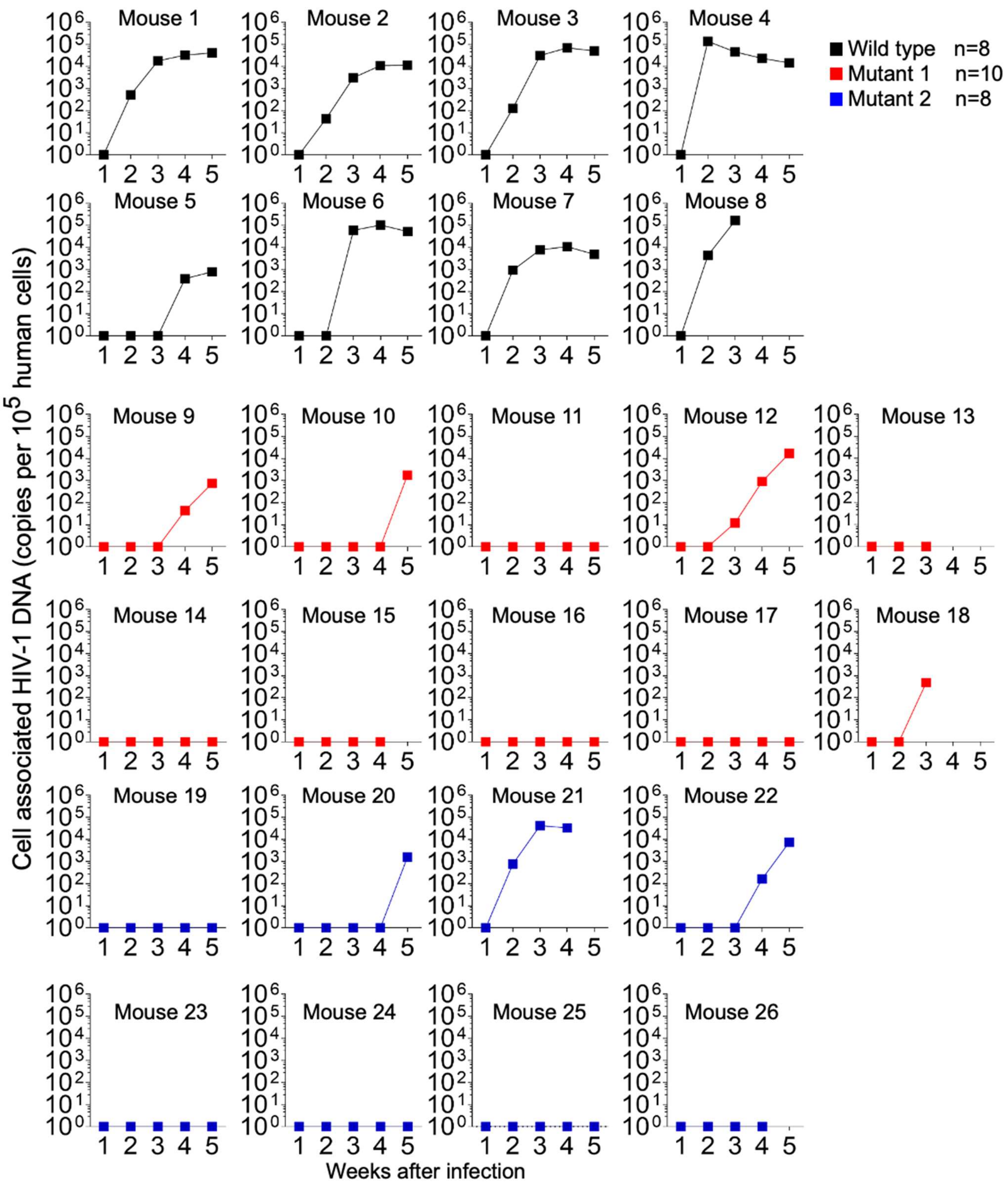

Figure S1. HIV-1 proviral DNA monitored longitudinally by qPCR.
